# Association of State-Level Medicaid Expansion With Treatment of Patients With Higher-Risk Prostate Cancer

**DOI:** 10.1001/jamanetworkopen.2020.15198

**Published:** 2020-10-07

**Authors:** Wen Liu, Michael Goodman, Christopher P. Filson

**Affiliations:** 1 Department of Urology, NYU Langone School of Medicine, New York, New York; 2 Rollins School of Public Health, Emory University, Atlanta, Georgia; 3 Department of Urology, Emory University School of Medicine, Atlanta, Georgia; 4 Winship Cancer Institute, Emory Healthcare, Atlanta, Georgia

## Abstract

**Importance:**

The Patient Protection and Affordable Care Act broadened insurance coverage, partially through voluntary state-based Medicaid expansion.

**Objective:**

To determine whether patients with higher-risk prostate cancer residing in Medicaid expansion states were more likely to receive treatment after expansion compared with patients in states electing not to pursue Medicaid expansion.

**Design, Setting, and Participants:**

This population-based cohort study included 15 332 patients diagnosed with higher-risk prostate cancer (ie, grade group >2; grade group 2 with prostate-specific antigen levels >10 ng/mL; or grade group 1 with prostate-specific antigen levels >20 ng/mL) from January 2010 to December 2016 aged 50 to 64 years who were candidates for definitive treatment. Patients residing in states that partially expanded Medicaid coverage before 2010 (ie, California and Connecticut) and those with diagnosis not confirmed by histology were excluded. Data were collected from the Surveillance, Epidemiology, and End Results Program. Data were analyzed between August and December 2019.

**Exposure:**

State-level Medicaid expansion status.

**Main Outcomes and Measures:**

Insurance status before and after expansion, treatment with prostatectomy or radiation therapy (including brachytherapy), treatment trends over time.

**Results:**

Of 15 332 patients, 7811 (50.9%) lived in expansion states (mean [SD] age, 59.1 [3.8] years; 5532 [71.9%] non-Hispanic White), and 7521 (49.1%) lived in nonexpansion states (mean [SD] age, 59.0 [3.9] years; 3912 [52.1%] non-Hispanic White). Residence in an expansion state was associated with higher pre-expansion levels of Medicaid coverage (292 [8.1%] vs 161 [3.8%]; odds ratio [OR], 2.12; 95% CI, 1.78 to 2.53) and lower likelihood of being uninsured (136 [3.2%] vs 38 [1.1%]; OR, 0.28; 95% CI, 0.15 to 0.54). After expansion, there was no difference in trends in treatment receipt between expansion and nonexpansion states (change, −0.39%; 95% CI, −0.11% to 0.28%; *P* = .25). Patients with private or Medicare coverage were more likely to receive treatment vs those with Medicaid or no coverage across racial/ethnic groups (eg, Black patients with coverage: OR, 2.30; 95% CI, 1.68 to 3.10; Black patients with no coverage: OR, 1.48; 95% CI, 1.09 to 2.00; *P* < .001). Medicaid patients were not more likely to be treated compared with those without insurance (737 [78.8%] vs 435 [79.5%]; OR, 0.97; 95% CI, 0.76 to 1.25).

**Conclusions and Relevance:**

In this cohort study, state-level expansion of Medicaid was associated with increased Medicaid coverage for men with higher-risk prostate tumors but did not appear to affect treatment patterns at a population level. This may be related to the finding that Medicaid coverage was not associated with increased treatment rates compared with those without insurance.

## Key Points

**Question:**

How is Medicaid expansion associated with the insurance status and treatment patterns among patients with higher-risk prostate cancer?

**Findings:**

In this cohort study that included 15 332 men newly diagnosed with higher-risk prostate cancer, Medicaid coverage increased after expansion but was not associated with higher treatment rates at the population level after expansion or compared with uninsured patients.

**Meaning:**

Although there was no difference in higher-risk prostate cancer treatment rates after Medicaid expansion in this study, it is important to continue addressing disparities in at-risk populations, given that insurance coverage alone is not sufficient to improve cancer care.

## Teaser

This cohort study evaluates whether patients with higher-risk prostate cancer residing in Medicaid expansion states were more likely to receive treatment after expansion compared with patients in states that did not pursue Medicaid expansion.

## Introduction

Amid major advances in medicine and technology, inequity in access to health care services persists. The Patient Protection and Affordable Care Act (ACA) increased health insurance coverage through multiple mechanisms, including permitting states to expand Medicaid eligibility at their discretion, with some states expanding prior to 2014 and some opting not to adopt expansion. ACA implementation took effect on January 1, 2014, and allowed states to expand coverage for childless adults younger than 65 years whose income was 133% of the federal poverty level or lower. To date, more than 20 million previously uninsured individuals have gained coverage, with the proportion of Americans without health insurance reaching a historic low.^1,2^ Newly covered individuals disproportionately belonged to minority racial/ethnic groups, were older, had poorer health, and were more likely to delay care due to cost.^3,4,5^ Racial disparities in all-cause mortality were reduced following increased insurance coverage.^1,4,6^ After ACA implementation, there were decreases in the proportion of uninsured patients among lower-income residents in expansion states^5,6^ along with increased insurance coverage and health care utilization for low-income adults.^7^

Increased insurance coverage has the potential to improve access to cancer care and possibly reduce persistent disparities in cancer survival based on race/ethnicity^8^ and socioeconomic status.^9^ These disparities are driven partly by a lack of health insurance, which affects receipt of cancer screening,^10^ resulting in higher rates of advanced malignant neoplasms.^11,12,13,14,15,16^ Medicaid expansion may also help at-risk men who otherwise would not undergo prostate cancer screening.^17^ Acknowledging that improved screening may risk possible overtreatment of indolent disease, contemporary practice emphasizes shared decision-making and often includes secondary screening tools and further imaging to minimize potential harms. The ability to successfully diagnose and definitively treat prostate cancer in younger and/or healthy men has considerable implications due to excellent survival rates.

However, even with improved access to care disparities in timely and appropriate treatment may result in differences in survival outcomes. An analysis of Surveillance, Epidemiology, and End Results (SEER; National Cancer Institute) data from 1992 to 2009 found evidence of a substantial difference in quality of surgical care for prostate cancer in Black patients, with not just longer treatment delay but also lower likelihood of receiving radical prostatectomy within 3 months of diagnosis compared with non-Hispanic White men.^18^ A similar study found that men younger than 65 years with nonpalpable prostate cancer were more likely to receive conservative management if they were Black and had no insurance—or even state Medicaid coverage—than if they had private insurance during times of economic hardship.^19^

We hypothesized that increasing access to care through Medicaid expansion would be associated with increased prostate cancer treatment at a population level for men diagnosed with higher-risk prostate tumors (Gleason grade group [GG] 3-5; GG 2 with prostate-specific antigen (PSA) level ≥10 ng/mL [to convert to micrograms per liter, multiply by 1.0]; or GG 1 with PSA level >20 ng/mL) that would result in definitive treatment.^20^ To test this, we used cancer registry data to compare patterns of prostate cancer treatment in states that did and did not expand Medicaid coverage. Demonstration of the downstream consequences of insurance expansion on prostate cancer treatment patterns would have important policy implications in terms of identifying mechanisms to decrease barriers to potentially life-saving interventions for men with prostate cancer.

## Methods

### Data Set

We used SEER data from January 2010 through December 2016.^21^ The SEER program reports information on cancer incidence and mortality and provides patient-level data on selected demographic and tumor-specific factors from 18 cancer registries, covering approximately 28% of the US population.^21^ Per the Common Rule, this study was deemed exempt from institutional review board oversight because it relied on publicly available deidentified data. Reporting of this study followed the Strengthening the Reporting of Observational Studies in Epidemiology (STROBE) reporting guideline.

### Study Population

The analytic cohort was created as displayed in eFigure 1 in the Supplement. We identified men aged 50 to 64 years newly diagnosed with prostate cancer during the 7-year study period, which extended from January 2010 to December 2016. We limited our analysis to SEER registries with statewide coverage and excluded patients residing in states that partially expanded Medicaid coverage prior to 2010 (ie, California and Connecticut). We excluded nonhistologically confirmed cases and cases from autopsy or death certificate reporting. We limited the analysis to patients with higher-risk tumors that are more appropriate for definitive cancer-directed treatment (ie, any biopsy with GG 3-5; GG 2 with PSA level ≥10 ng/mL; or GG 1 with PSA level >20 ng/mL).

### Exposures and Outcomes

The primary exposure was state Medicaid expansion status. On January 1, 2014, 5 states with SEER data expanded Medicaid coverage (ie, Hawaii, Iowa, New Mexico, Kentucky, and New Jersey) and 3 did not (ie, Georgia, Louisiana, and Utah). We assessed whether Medicaid expansion was associated with a change in the proportion of patients with Medicaid coverage and lack of insurance. Our primary outcome of interest was treatment with radical prostatectomy or radiation therapy (including brachytherapy). The main exposures were state-level expansion status and timing of diagnosis (pre-expansion vs postexpansion). Patient-level covariates of interest included patient age (continuous), race/ethnicity as designated by SEER cancer registrars (non-Hispanic White, non-Hispanic Black, Hispanic, or other), marital status (single; married; separated, divorced, or widowed; or unknown), insurance status (uninsured, Medicaid, or insured), PSA level (continuous), and biopsy grade group (1, 2, 3, or 4-5).

### Statistical Analysis

We performed bivariate analyses to evaluate unadjusted associations between residence in a Medicaid expansion state (vs nonexpansion state) and receipt of treatment with various demographic and clinical patient characteristics. Multivariable logistic regression models were fit to examine the association between factors of interest with Medicaid coverage, lack of insurance, and the receipt of treatment. Models assessing insurance coverage adjusted for age (continuous), race/ethnicity (categorical), and marital status (categorical) and included an interaction term for expansion status and time before or after expansion. Models assessing receipt of treatment included those covariates plus insurance status. Models were adjusted for clustering at the state level with a robust variance estimator. The variance inflation factor was assessed for all models without multicollinearity observed between the variables. The results of the logistic regression analyses were expressed as adjusted odds ratios (aORs) and corresponding 95% CIs. Estimated probabilities with 95% CIs were generated using postestimation commands.

Trends over time were assessed using an interrupted time-series regression model with a multiple group comparison (eAppendix in the Supplement).^22^ The interruption point was assumed to coincide with Medicaid expansion on January 1, 2014. The main analysis compared trends in states with and without Medicaid expansion. Newey-West SEs were used to address autocorrelation and possible heteroscedasticity. From these models, the slope over time for each group was estimated before and after the intervention, and the difference-in-differences of slopes before and after the intervention was then calculated. Finally, the slopes of changes in treatment receipt were plotted for easier visualization of results.

All analyses were performed using Stata version 16.1 (StataCorp). Data were analyzed between August and December 2019. A 2-sided *P* < .05 was considered statistically significant for all analyses. Patients with missing data (accounting for approximately 10% of the total patients) were excluded to minimize bias that can be associated with imputation.

## Results

The sociodemographic and clinical characteristics of the study cohort are outlined in Table 1. We identified 15 332 men diagnosed with histologically confirmed high-risk prostate cancer between January 1, 2010, and December 31, 2016. The mean age at diagnosis was 59 years for patients in both expansion and nonexpansion states (SD, 3.8 and 3.9, respectively). There were 7811 patients (50.9%) diagnosed in expansion states (mean [SD] age, 59.1 [3.8] years; 5532 [71.9%] non-Hispanic White) and 7521 (49.1%) in nonexpansion states (mean [SD] age, 59.0 [3.9] years; 3912 [52.1%] non-Hispanic White). Most men in expansion states lived in New Jersey (3567 [45.7%]), and most men in nonexpansion states lived in Georgia (4352 [57.9%]). A greater proportion of men in nonexpansion states than in expansion states were non-Hispanic Black men (3352 [44.7%] vs 1125 [14.6%]; *P* < .001). Overall, 13 210 patients (86.2%) were treated with either radical prostatectomy or radiation therapy (eTable in the Supplement).

**Table 1.** Demographic Characteristics of Analytic Cohort

Characteristic	Patients, No. (%)		*P* value
	Nonexpansion state (n = 7521)	Expansion state (n = 7811)	
Age, mean (SD), y	59.0 (3.9)	59.1 (3.8)	.12
Year of diagnosis			
2010	1056 (14.0)	1029 (13.2)	.007
2011	1035 (13.8)	1085 (13.9)	
2012	995 (13.2)	1039 (13.3)	
2013	1031 (13.7)	1061 (13.6)	
2014	974 (13.0)	1164 (14.9)	
2015	1125 (15.0)	1189 (15.2)	
2016	1305 (17.4)	1244 (15.9)	
State			
Hawaii	0	500 (6.4)	<.001
Iowa	0	1595 (20.4)	
New Mexico	0	568 (7.3)	
Utah	859 (11.4)	0	
Georgia	4352 (57.9)	0	
Kentucky	0	1581 (20.2)	
Louisiana	2310 (30.7)	0	
New Jersey	0	3567 (45.7)	
Race/ethnicity^a^			
Non-Hispanic			<.001
White	3912 (52.1)	5532 (71.9)	
Black	3352 (44.7)	1125 (14.6)	
Hispanic/Latino	166 (2.2)	539 (7.0)	
Other	78 (1.0)	494 (6.4)	
Marital status			
Single	1170 (15.6)	994 (12.7)	<.001
Married or domestic partner	4518 (60.1)	5011 (64.2)	
Separated, divorced, or widowed	992 (13.2)	902 (11.6)	
Missing	841 (11.2)	904 (11.6)	
Insurance status^b^			
Insured			<.001
Private or Medicare	6094 (87.7)	6378 (91.1)	
Medicaid	482 (6.9)	453 (6.5)	
Uninsured	373 (5.4)	174 (2.5)	
Tumor stage^c^			
T1	4708 (62.6)	4228 (54.1)	<.001
T2	1815 (24.1)	2285 (29.3)	
T3-T4	163 (2.2)	222 (2.8)	
PSA, ng/mL			
<10.0	3886 (51.7)	4169 (53.4)	.08
10.0-19.9	2327 (30.9)	2362 (30.2)	
>20.0	1308 (17.4)	1280 (16.4)	
Gleason Score on biopsy^d^			
3 + 3 = 6; GG1	219 (2.9)	252 (3.2)	.002
3 + 4 = 7; GG2	1384 (18.4)	1310 (16.8)	
4 + 3 = 7; GG3	3031 (40.3)	3054 (39.1)	
≥4 + 4 = 8; GG4-5	2887 (38.4)	3195 (40.9)	

Abbreviations: GG, grade group; PSA, prostate-specific antigen.

SI conversion factor: To convert PSA to micrograms per liter, multiply by 1.0.

^a^ Missing in 134 cases.

^b^ Missing in 1378 cases.

^c^ Missing in 1911 cases.

^d^ Gleason Score represents the most common pattern plus the second most common pattern noted on biopsy.

The association between patient factors and insurance type is displayed in Table 2. Non-Hispanic White men were significantly less likely to be covered by Medicaid compared with non-Hispanic Black (367 [3.9%] vs 477 [10.7%]; aOR, 2.30; 95% CI, 1.68-3.16) and Hispanic/Latino men (57 [8.1%]; aOR, 1.86; 95% CI 1.04-3.31). Similar findings were noted for being uninsured; non-Hispanic Black men (246 [5.5%]; aOR, 1.48; 95% CI, 1.09-2.00) and Hispanic/Latino men (62 [8.8%]; aOR, 4.89; 95% CI, 2.97-8.08) were more likely to have no insurance coverage than non-Hispanic White men (227 [2.4%]). Single men were more likely than married men to have Medicaid (406 [18.8%] vs 274 [2.9%]; aOR, 6.43; 95% CI, 4.78-8.66) or be uninsured (189 [8.7%] vs 212 [2.2%]; aOR, 3.79; 95% CI, 3.01-4.78). In expansion states, there was an increased proportion of men with Medicaid coverage after expansion compared with before expansion (292 [8.1%] vs 161 [3.8%]; aOR, 2.12; 95% CI, 1.78-2.53). In these states, there was also a significant decrease in the uninsured proportion after expansion compared with before expansion (38 [1.1%] vs 136 [3.2%]; aOR, 0.28; 95% CI, 0.15-0.54). Nonexpansion states did not experience any significant change in the proportion of those covered with Medicaid postexpansion vs pre-expansion (226 [6.6%] vs 256 [6.2%]; aOR, 0.98; 95% CI, 0.88-1.10) but did see a decrease in the proportion without insurance (136 [4.0%] vs 237 [5.8%]; aOR, 0.62; 95% CI, 0.52-0.75).

**Table 2.** Factors Associated With Medicaid Coverage or Uninsured Status for Analytic Cohort

Factor	Medicaid coverage		Uninsured	
	No. (%)	Adjusted OR (95% CI)^a^	No. (%)	Adjusted OR (95% CI)^a^
**Demographic characteristics**				
Race/ethnicity				
Non-Hispanic				
White	367 (3.9)	1 [Reference]	227 (2.4)	1 [Reference]
Black	477 (10.7)	2.30 (1.68-3.16)	246 (5.5)	1.48 (1.09-2.00)
Hispanic/Latino	57 (8.1)	1.86 (1.04-3.31)	62 (8.8)	4.89 (2.97-8.08)
Other	32 (5.6)	1.37 (0.67-2.80)	11 (1.9)	1.05 (0.46-2.41)
Marital status				
Married or domestic partner	274 (2.9)	1 [Reference]	212 (2.2)	1 [Reference]
Single	406 (18.8)	6.43 (4.78-8.66)	189 (8.7)	3.79 (3.01-4.78)
Separated, divorced, or widowed	200 (10.6)	3.66 (2.53-5.28)	121 (6.4)	2.82 (2.06-3.87)
Missing	55 (3.2)	1.03 (0.80-1.35)	25 (1.4)	0.60 (0.46-0.79)
**Expansion status and diagnosis date**				
Expansion state				
Pre-expansion	161 (3.8)	1 [Reference]	136 (3.2)	1 [Reference]
Postexpansion	292 (8.1)	2.12 (1.78-2.53)	38 (1.1)	0.28 (0.15-0.54)
Nonexpansion state				
Pre-expansion	256 (6.2)	1 [Reference]	237 (5.8)	1 [Reference]
Postexpansion	226 (6.6)	0.98 (0.88-1.10)	136 (4.0)	0.62 (0.52-0.75)

Abbreviation: OR, odds ratio.

^a^ Age-adjusted model based on 15 198 men with complete data.

**Figure 1.**
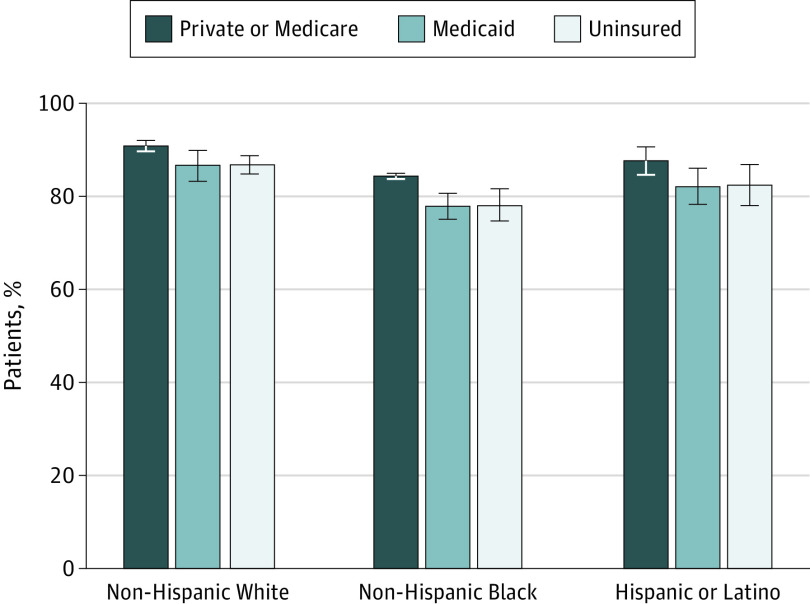
Treatment of High-Risk Prostate Cancer Based on Race/Ethnicity and Insurance Status This figure displays the estimated proportion of patients with high-risk prostate cancer who were treated, based on race/ethnicity and insurance status. The values are based on estimated margins from models using a cohort of 13 912 men with complete data adjusting for patient age, prostate-specific antigen level, biopsy Gleason score, marital status, race/ethnicity, insurance status, state-level expansion, and timing of diagnosis (pre-expansion vs postexpansion). Whiskers indicate 95% CIs.

The associations between race, insurance status, expansion status, and treatment receipt are depicted in Table 3. Compared with White men, non-Hispanic Black men (aOR, 0.65; 95% CI, 0.52-0.81) and Hispanic/Latino men (aOR, 0.71; 95% CI, 0.54-0.94) were less likely to undergo prostatectomy or radiation therapy. Men with private insurance or Medicare were more likely to receive treatment than men without insurance (aOR, 1.52; 95% CI, 1.25-1.86). Figure 1 demonstrates the estimated proportions of patients with prostate cancer receiving treatment based on race/ethnicity and insurance status. Patients who had private or Medicare insurance coverage were most likely to undergo treatment across all racial/ethnic groups: 91.0% (95% CI, 89.7%-92.1%) for non-Hispanic White patients, 84.5% (95% CI, 83.9%-85.1%) for non-Hispanic Black patients, and 87.7% (95% CI, 84.7%-90.8%) for Hispanic/Latino patients. In addition, patients in all racial/ethnic groups who had Medicaid coverage or no insurance had similar treatment rates.

**Table 3.** Race/Ethnicity, Insurance Status, State-Level Medicaid Expansion, and Treatment of High-Risk Prostate Cancer

Factor	Treatment with prostatectomy or radiation therapy	
	No. (%)	Adjusted OR (95% CI)^a^
Demographic Characteristics		
Race/ethnicity^b^		
Non-Hispanic		
White	8400 (89.0)	1 [Reference]
Black	3652 (81.6)	0.65 (0.52-0.81)
Hispanic/Latino	594 (84.3)	0.71 (0.54-0.94)
Other	507 (88.6)	0.92 (0.66-1.28)
Insurance status^c^		
Uninsured	435 (79.5)	1 [Reference]
Medicaid	737 (78.8)	0.97 (0.76-1.25)
Private/Medicare	11 144 (89.4)	1.52 (1.25-1.86)
**Expansion status and diagnosis date**		
Expansion state		
Pre-expansion	3725 (88.6)	1 [Reference]
Postexpansion	3169 (88.1)	0.95 (0.83-1.08)
Nonexpansion state		
Pre-expansion	3424 (83.2)	1 [Reference]
Postexpansion	2882 (84.7)	1.44 (1.16-1.80)

Abbreviation: OR, odds ratio.

^a^ Based on cohort of 13 912 men with complete data after adjustment for patient age, prostate-specific antigen level, biopsy Gleason score, marital status, and covariates.

^b^ Missing in 134 cases.

^c^ Missing in 1378 cases.

**Figure 2.**
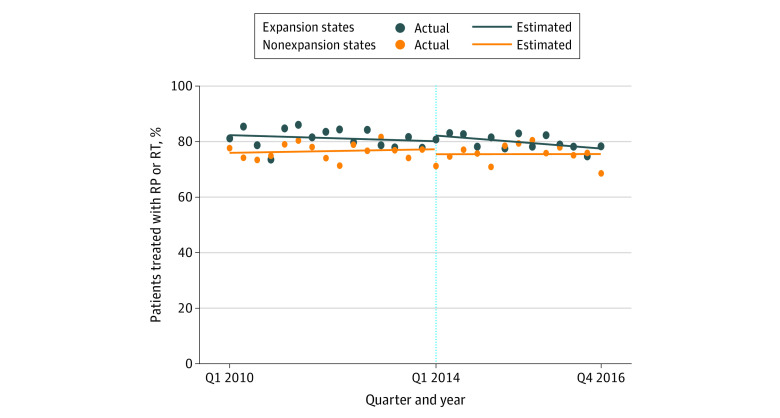
Time Trends in Treatment of Patients With High-Risk Prostate Cancer Based on State-Level Medicaid Expansion This figure demonstrates the trends in quarterly proportions of patients with high-risk prostate cancer residing in expansion states (blue dots) and nonexpansion states (orange dots) who underwent treatment with radical prostatectomy (RP) or radiation therapy (RT). The vertical dotted line at Q1 2014 indicates when Medicaid expansion was approved in expansion states.

Trends in treatment of patients with high-risk prostate cancer based on state-level expansion are demonstrated in Figure 2. Before expansion, there was no significant change over time for either group (expansion states: β = −0.14%; 95% CI, −0.55% to 0.26%; *P* = .48; nonexpansion states: β = 0.10%; 95% CI, −0.20% to 0.37%; *P* = .54), and the trends were essentially parallel (change [calculated as β for expansion states − β for nonexpansion states], −0.23%; 95% CI, −0.72% to 0.26%; *P* = .35). Following expansion, there was a clinically insignificant but statistically significant decrease in the proportion treated in expansion states (β = −0.38%; 95% CI, −0.66% to 0.11%; *P* = .01) and no significant change in nonexpansion states (β = 0.01%; 95% CI, −0.61% to 0.62%; *P* = .99). However, there was no significant postexpansion difference between these trends (change, −0.39%; 95% CI, −0.11% to 0.28%; *P* = .25).

## Discussion

Our analysis produced 3 major findings. First, we showed that patients with prostate cancer who had clinically significant tumors and resided in states that expanded Medicaid eligibility after January 2014 were significantly more likely to have Medicaid coverage after expansion and that similar changes in Medicaid coverage were not seen in nonexpansion states. Second, despite increases in Medicaid coverage at the patient level, state-level expansion of Medicaid coverage was not associated with any significant short-term change in treatment trends among men with clinically significant prostate tumors at the population level. Finally, we found that patients with Medicaid insurance had similar treatment rates as those without any insurance coverage, which persisted across racial/ethnic groups.

Insurance coverage may improve access to cancer screening and care and mitigate cancer-specific disparities in timing of diagnosis and treatment receipt. Observational studies have shown that Medicare-ineligible insured men with high-risk prostate cancer were less likely to present with metastatic disease and more likely to receive definitive therapy and experience better survival compared with those without insurance.^23^ The seminal randomized clinical trial in Oregon assessed the impact of Medicaid expansion and demonstrated that patients who gained Medicaid coverage were more likely to undergo certain cancer screening tests.^24^ In Kentucky, expansion of Medicaid coverage was associated with a greater proportion of women being diagnosed with early-stage disease and improvements in the quality of breast cancer care that was delivered.^25^

Although these examples suggest state-level Medicaid expansion may improve cancer care at a population level, our analysis did not demonstrate similar results for patients with prostate cancer after 2014 associated with treatment of significant prostate tumors. There are likely multiple explanations for this finding. First, only approximately 15% of US residents aged 19 to 64 years have Medicaid coverage,^26^ so expansion at the state level results in a comparatively small number of at-risk men gaining coverage. Second, more than 85% of our cohort received tumor-directed treatment for their prostate cancer, leaving relatively little room for improvement. Third, although insurance coverage theoretically creates access for patients who otherwise would not be able to afford cancer treatment, barriers to adequate specialist care persists for Medicaid patients.

Data from the National Health Interview Survey demonstrated that patients who gained insurance coverage after Medicaid expansion still had to battle prolonged wait times for appointments and difficulties finding a clinician to meet their health care needs.^27,28^ A 2018 survey of adults residing in California demonstrated that Medicaid recipients had poor access to primary care and specialty physicians compared with those covered by employer-based insurance.^29^ In this study, more than 20% of Medicaid recipients tried to go to a specialist who did not accept their insurance, and more than 25% could not get an appointment in a timely manner. Regarding prostate cancer, low-income patients undergo different treatments based on whether they are managed at county vs private hospitals.^30^ Other studies have shown that men with Medicaid coverage were less likely to be treated at hospitals offering robotic-assisted radical prostatectomy.^31^ On the other hand, disadvantaged patients with cancer have experienced more equitable care in single-payer settings, such as the Veterans Health Administration.^32,33^ Clearly, Medicaid coverage alone does not completely remove barriers to adequate cancer care for low-income patients with prostate cancer. Nevertheless, although we did not observe large shifts in treatment for patients with prostate cancer in Medicaid expansion states, there are likely benefits for this population beyond treatment. Sammon et al^17^ reported that disparities in PSA screening rates between higher- and lower-income men narrowed in Medicaid expansion states, possibly reflecting better access to preventive services with increased insurance coverage. Nevertheless, depending on the methods and patient population, reports show the proportion of men who undergo annual PSA screening ranges from 22% to 30%.^17,34^

It is imperative to continue dedicating efforts to improve outcomes and survival for patients who may be the most marginalized—the uninsured. Expansion of insurance coverage will not accomplish this alone. Furthermore, efforts to improve prostate cancer care for those most at risk of adverse outcomes should also focus on Medicaid patients. Among a group of uninsured patients with prostate cancer previously managed by a disease-specific, state-funded treatment program, men who left the program after gaining comprehensive insurance coverage had inferior physical health compared with those who stayed in the program.^35^ Although some improvement is required for patients with prostate cancer, Medicaid expansion has considerable benefits at a population level with regards to financial security, chronic disease care, and overall well-being.^10^ Ongoing policy efforts must focus on ensuring equitable access to prostate cancer and urologic care for those covered by the Medicaid program.

### Limitations

This study should be interpreted in the context of its limitations. First, the treatment status for prostate cancer patients in SEER is not 100% sensitive, particularly for those receiving radiation therapy. However, we did not observe major differences in types of treatments received based on insurance status, so we should not anticipate a major bias in our results related to this fact. Second, because the proportion of patients with Medicaid or no insurance was relatively small, we may have not been able to observe subtle changes in treatment rates due to insufficient sample size. Furthermore, due to the geographic basis of the SEER cancer registry, we are unable to account for patients who change residence out of areas captured during the time frame of the study.

## Conclusions

In this cohort study, expansion of Medicaid coverage at the state level in 2014 was associated with increased Medicaid coverage and a lower proportion of uninsured men among patients with clinically significant prostate tumors. However, Medicaid expansion was not associated with changes in treatment receipt in this population.

## Notes

Supplement. **eAppendix. **Regression Model to Assess Trends Over Time **eFigure. **Generation of Analytic Cohort **eTable. **Association With Additional Factors and Treatment of Men With High-Risk Prostate Cancer

## References

[1] Artiga S, Orgera K, Pham O Disparities in health and health care: five key questions and answers. Kaiser Family Foundation. Published March 4, 2020. Accessed September 8, 2020. https://www.kff.org/disparities-policy/issue-brief/disparities-in-health-and-health-care-five-key-questions-and-answers/

[2] Obama B United States health care reform: progress to date and next steps. JAMA. 2016;316(5):525-532. doi:10.1001/jama.2016.9797 27400401 PMC5069435

[3] Akinyemiju T, Jha M, Moore JX, Pisu M Disparities in the prevalence of comorbidities among US adults by state Medicaid expansion status. Prev Med. 2016;88:196-202. doi:10.1016/j.ypmed.2016.04.009 27095325 PMC4902718

[4] Sommers BD, Baicker K, Epstein AM Mortality and access to care among adults after state Medicaid expansions. N Engl J Med. 2012;367(11):1025-1034. doi:10.1056/NEJMsa1202099 22830435

[5] Sommers BD, Gunja MZ, Finegold K, Musco T Changes in self-reported insurance coverage, access to care, and health under the Affordable Care Act. JAMA. 2015;314(4):366-374. doi:10.1001/jama.2015.8421 26219054

[6] Sommers BD, Blendon RJ, Orav EJ, Epstein AM Changes in utilization and health among low-income adults after Medicaid expansion or expanded private insurance. JAMA Intern Med. 2016;176(10):1501-1509. doi:10.1001/jamainternmed.2016.4419 27532694

[7] Wherry LR, Miller S Early coverage, access, utilization, and health effects associated with the Affordable Care Act Medicaid expansions: a quasi-experimental study. Ann Intern Med. 2016;164(12):795-803. doi:10.7326/M15-2234 27088438 PMC5021068

[8] Tewari A, Horninger W, Pelzer AE, Factors contributing to the racial differences in prostate cancer mortality. BJU Int. 2005;96(9):1247-1252. doi:10.1111/j.1464-410X.2005.05824.x 16287439

[9] Doubeni CA, Jambaulikar GD, Fouayzi H, Neighborhood socioeconomic status and use of colonoscopy in an insured population—a retrospective cohort study. PLoS One. 2012;7(5):e36392. doi:10.1371/journal.pone.0036392 22567154 PMC3342210

[10] Sommers BD, Gawande AA, Baicker K Health insurance coverage and health—what the recent evidence tells us. N Engl J Med. 2017;377(6):586-593. doi:10.1056/NEJMsb1706645 28636831

[11] Potosky AL, Breen N, Graubard BI, Parsons PE The association between health care coverage and the use of cancer screening tests: results from the 1992 National Health Interview Survey. Med Care. 1998;36(3):257-270. Erratum in: Med Care 1998 Oct;36. 10:1470. doi:10.1097/00005650-199803000-00004 9520952

[12] Halpern MT, Bian J, Ward EM, Schrag NM, Chen AY Insurance status and stage of cancer at diagnosis among women with breast cancer. Cancer. 2007;110(2):403-411. doi:10.1002/cncr.22786 17562557

[13] Breen N, Wagener DK, Brown ML, Davis WW, Ballard-Barbash R Progress in cancer screening over a decade: results of cancer screening from the 1987, 1992, and 1998 National Health Interview Surveys. J Natl Cancer Inst. 2001;93(22):1704-1713. doi:10.1093/jnci/93.22.1704 11717331

[14] DeVoe JE, Fryer GE, Phillips R, Green L Receipt of preventive care among adults: insurance status and usual source of care. Am J Public Health. 2003;93(5):786-791. doi:10.2105/AJPH.93.5.786 12721145 PMC1447840

[15] Roetzheim RG, Pal N, Gonzalez EC, Ferrante JM, Van Durme DJ, Krischer JP Effects of health insurance and race on colorectal cancer treatments and outcomes. Am J Public Health. 2000;90(11):1746-1754. doi:10.2105/AJPH.90.11.1746 11076244 PMC1446414

[16] Roetzheim RG, Pal N, Tennant C, Effects of health insurance and race on early detection of cancer. J Natl Cancer Inst. 1999;91(16):1409-1415. doi:10.1093/jnci/91.16.1409 10451447

[17] Sammon JD, Serrell EC, Karabon P, Prostate cancer screening in early Medicaid expansion states. J Urol. 2018;199(1):81-88. doi:10.1016/j.juro.2017.07.083 28765069

[18] Schmid M, Meyer CP, Reznor G, Racial differences in the surgical care of Medicare beneficiaries with localized prostate cancer. JAMA Oncol. 2016;2(1):85-93. doi:10.1001/jamaoncol.2015.3384 26502115 PMC5018381

[19] Weiner AB, Conti RM, Eggener SE National economic conditions and patient insurance status predict prostate cancer diagnosis rates and management decisions. J Urol. 2016;195(5):1383-1389. doi:10.1016/j.juro.2015.12.071 26707507

[20] American Urological Association Clinically localized prostate cancer: AUA/ASTRO/SUO guideline (2017). Accessed September 8, 2020. https://www.auanet.org/guidelines/prostate-cancer-clinically-localized-guideline

[21] National Cancer Institute Surveillance, Epidemiology, and End Results Program. Accessed September 10, 2020. http://www.seer.cancer.gov

[22] Linden A. Conducting interrupted time-series analysis for single- and multiple-group comparisons. Stata Journal. 2015;15(2):480-500. doi:10.1177/1536867X1501500208

[23] Mahal BA, Aizer AA, Ziehr DR, The association between insurance status and prostate cancer outcomes: implications for the Affordable Care Act. Prostate Cancer Prostatic Dis. 2014;17(3):273-279. doi:10.1038/pcan.2014.23 24980272

[24] Wright BJ, Conlin AK, Allen HL, Tsui J, Carlson MJ, Li HF What does Medicaid expansion mean for cancer screening and prevention? results from a randomized trial on the impacts of acquiring Medicaid coverage. Cancer. 2016;122(5):791-797. doi:10.1002/cncr.29802 26650571 PMC6193753

[25] Ajkay N, Bhutiani N, Huang B, Early impact of Medicaid expansion and quality of breast cancer care in Kentucky. J Am Coll Surg. 2018;226(4):498-504. doi:10.1016/j.jamcollsurg.2017.12.041 29449123

[26] Kaiser Family Foundation Medicaid enrollment by age, fiscal year 2013. Accessed September 8, 2020. https://www.kff.org/medicaid/state-indicator/medicaid-enrollment-by-age/

[27] Miller S, Wherry LR Health and access to care during the first 2 years of the ACA Medicaid expansions. N Engl J Med. 2017;376(10):947-956. doi:10.1056/NEJMsa1612890 28273021

[28] Selden TM, Lipton BJ, Decker SL Medicaid expansion and marketplace eligibility both increased coverage, with trade-offs in access, affordability. Health Aff (Millwood). 2017;36(12):2069-2077. doi:10.1377/hlthaff.2017.0830 29200332

[29] Alcalá HE, Roby DH, Grande DT, McKenna RM, Ortega AN Insurance type and access to health care providers and appointments under the Affordable Care Act. Med Care. 2018;56(2):186-192. doi:10.1097/MLR.0000000000000855 29271819

[30] Parsons JK, Kwan L, Connor SE, Miller DC, Litwin MS Prostate cancer treatment for economically disadvantaged men: a comparison of county hospitals and private providers. Cancer. 2010;116(5):1378-1384. doi:10.1002/cncr.24856 20101733

[31] Kim SP, Boorjian SA, Shah ND, Disparities in access to hospitals with robotic surgery for patients with prostate cancer undergoing radical prostatectomy. J Urol. 2013;189(2):514-520. doi:10.1016/j.juro.2012.09.033 23253307

[32] Rhoads KF, Patel MI, Ma Y, Schmidt LA How do integrated health care systems address racial and ethnic disparities in colon cancer? J Clin Oncol. 2015;33(8):854-860. doi:10.1200/JCO.2014.56.8642 25624437 PMC4348634

[33] Daskivich TJ, Fan KH, Koyama T, Prediction of long-term other-cause mortality in men with early-stage prostate cancer: results from the Prostate Cancer Outcomes Study. Urology. 2015;85(1):92-100. doi:10.1016/j.urology.2014.07.003 25261048 PMC4275422

[34] Kearns JT, Holt SK, Wright JL, Lin DW, Lange PH, Gore JL PSA screening, prostate biopsy, and treatment of prostate cancer in the years surrounding the USPSTF recommendation against prostate cancer screening. Cancer. 2018;124(13):2733-2739. doi:10.1002/cncr.31337 29781117

[35] Nabhani JA, Kuang R, Liu H, Kwan L, Litwin MS Health changes in low income men transitioning from a state funded prostate cancer program to comprehensive insurance. J Urol. 2018;200(1):74-81. doi:10.1016/j.juro.2018.01.084 29425802

