# Supplementary material for: Association of State-Level Medicaid Expansion With Treatment of Patients With Higher-Risk Prostate Cancer
Source: JAMA Netw Open. 2020 Oct 7;3(10):e2015198. doi: 10.1001/jamanetworkopen.2020.15198 (PMC7542300; doi:10.1001/jamanetworkopen.2020.15198)
Supplement: Supplement. — eAppendix. Regression Model to Assess Trends Over Time eFigure. Generation of Analytic Cohort eTable. Association With Additional Factors and Treatment of Men With High-Risk Prostate Cancer [file jamanetwopen-e2015198-s001.pdf]

Supplemental Online Content

Liu W, Goodman M, Filson CP. Association of state-level Medicaid expansion with treatment of patients with higher-risk prostate cancer. *JAMA Netw Open*. 2020;3(10):e2015198. doi:10.1001/jamanetworkopen.2020.15198

eAppendix. Regression Model to Assess Trends Over Time
eFigure. Generation of Analytic Cohort
eTable. Association With Additional Factors and Treatment of Men With High-Risk Prostate Cancer

This supplemental material has been provided by the authors to give readers additional information about their work.

© 2020 Liu W et al. *JAMA Network Open.*

eAppendix. Regression Model to Assess Trends Over Time

$$Y = \beta_0 + \beta_1\, expand + \beta_2\, pre\text{-}time + \beta_3\, post\text{-}time + \beta_4\, expand{*}pre\text{-}time + \beta_5\, expand{*}post\text{-}time$$

where β_0 is the intercept, *expand* is the variable indicating the expansion (vs. non-expansion) status; *pre-time* and *post-time* are slopes before and after 2014, and *expand*pre-time* and *expand*post-time* are interaction terms reflecting the difference in slopes between expansion and non-expansion states. The analysis initially compared trends in states with and without Medicaid expansion (i.e., slopes β_{expand} and $\beta_{noexpand}$). The slope over time for each group was then estimated before and after the intervention ($\Delta_{pre\text{-}2014 \text{ or } post\text{-}2014} = \beta_{expand} - \beta_{noexpand}$). Finally, the difference-in-differences of slopes before and after the intervention was calculated ($\Delta_{overall} = \Delta_{pre\text{-}2014} - \Delta_{post\text{-}2014}$).

© 2020 Liu W et al. *JAMA Network Open.*

eFigure. Generation of Analytic Cohort

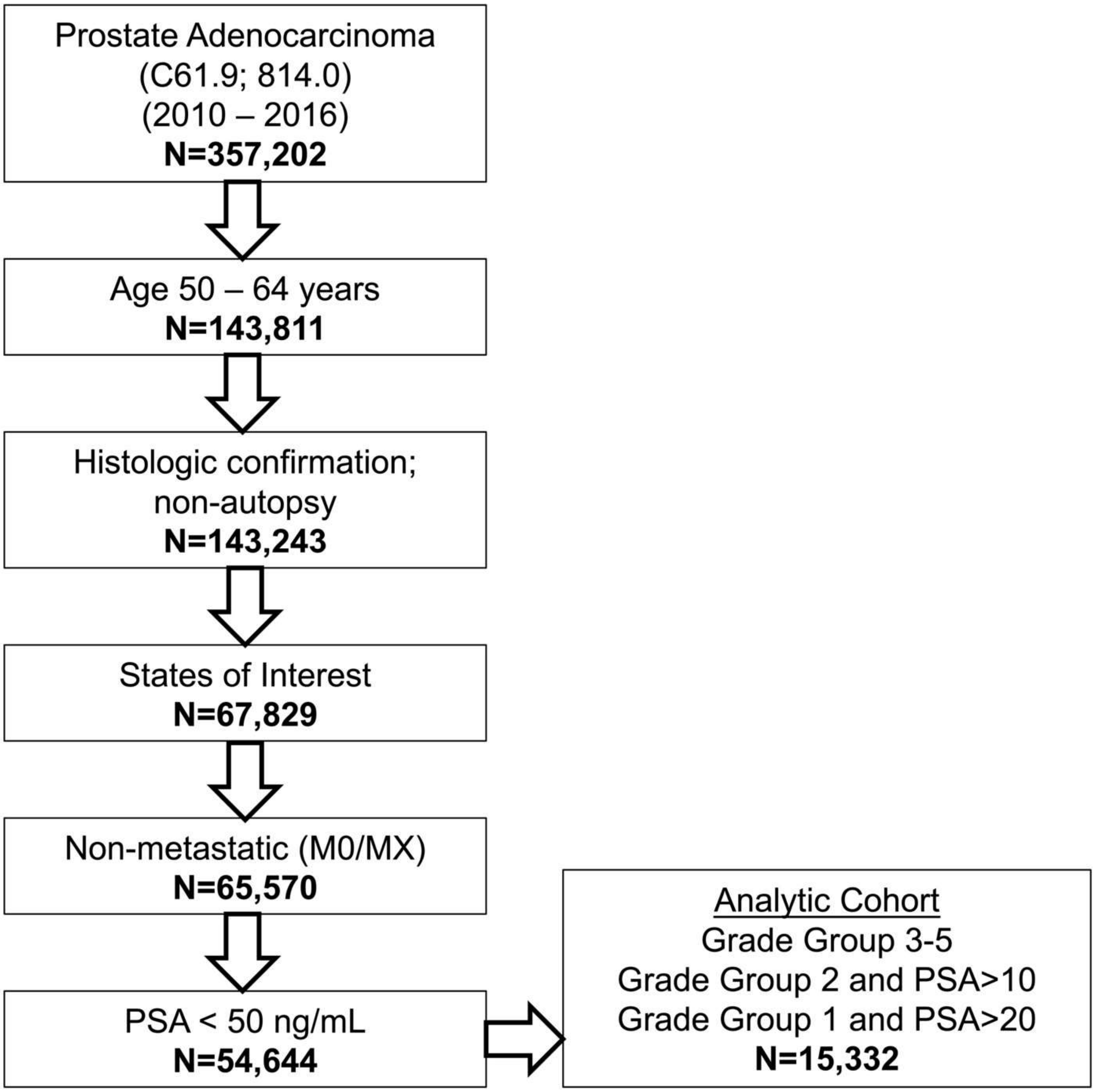

© 2020 Liu W et al. *JAMA Network Open.*

eTable. Association With Additional Factors and Treatment of Men With High-Risk Prostate Cancer

Covariate	Received Treatment (n=13,210)	No Treatment (n=2,122)	p
Age (years) (mean, SD)	59.1 (3.8)	59.2 (3.8)	.055
Year of Diagnosis			.042
2010	1,767 (84.8)	318 (15.3)	
2011	1,842 (86.9)	278 (13.1)	
2012	1,772 (87.1)	262 (12.9)	
2013	1,778 (85.0)	314 (15.0)	
2014	1,834 (85.8)	304 (14.2)	
2015	2,028 (87.6)	286 (12.4)	
2016	2,189 (85.9)	360 (14.1)	
State			<.001
Hawaii	434 (86.8)	66 (13.2)	
Iowa	1,465 (91.9)	130 (8.2)	
New Mexico	453 (79.8)	115 (20.3)	
Utah	767 (89.3)	92 (10.7)	
Georgia	3,631 (83.4)	721 (16.6)	
Kentucky	1,388 (87.8)	193 (12.2)	
Louisiana	1,908 (82.6)	402 (17.4)	
New Jersey	3,164 (88.7)	403 (11.3)	
Marital Status			<.001
Single	1,785 (82.5)	379 (17.5)	
Married/Domestic Partner	8,606 (90.3)	923 (9.7)	
Separated/Divorced/Widowed	1,632 (86.1)	262 (13.8)	
Missing	1,187 (68.0)	558 (32.0)	
Tumor Stage[a]			.001
T1	7,576 (84.7)	1,360 (15.2)	
T2	3,547 (86.5)	553 (13.5)	
T3-T4	309 (80.3)	76 (19.7)	
PSA (ng/mL)			<.001
<10.0	7,184 (89.2)	871 (10.8)	
10.0 – 19.9	4,003 (85.4)	686 (14.6)	
20.0+	2,023 (78.2)	565 (21.8)	
Gleason Score on Biopsy			<.001
3+3=6 (GG1)	321 (68.2)	150 (31.9)	
3+4=7 (GG2)	2,292 (85.1)	402 (14.9)	
4+3=7 (GG3)	5,366 (88.2)	719 (11.8)	
4+4=8 or greater (GG4-5)	5,231 (86.1)	851 (14.0)	

[a]Missing in 1,911 cases.

© 2020 Liu W et al. *JAMA Network Open.*
